# The Relationship Between Anti-Hypertensive Drugs and Cancer: Anxiety to be Resolved in Urgent

**DOI:** 10.3389/fphar.2020.610157

**Published:** 2020-12-14

**Authors:** Rong Yang, Yonggang Zhang, Xiaoyang Liao, Yi Yao, Chuanying Huang, Lixia Liu

**Affiliations:** ^1^ Department of International Medical Center/Ward of General Practice, West China Hospital, Sichuan University, Chengdu, China; ^2^ Department of Periodical Press and National Clinical Research Center for Geriatrics, West China Hospital, Sichuan University, Chengdu, China; ^3^ Health Management Center, West China Hospital, Sichuan University, Chengdu, China

**Keywords:** anti-hypertensive drugs, cancer, mechanisms, cancer risk, adjuvant therapy

## Abstract

Hypertension is the prevailing independent risk factor for cardiovascular disease worldwide. Anti-hypertensive drugs are the common and effective cure for lowering blood pressure in patients with hypertension. However, some large-scale clinical studies have pointed out that long-term ingestion of some oral anti-hypertensive drugs was associated with risks of incident cancer and the survival time. In contrast, other studies argue that anti-hypertensive drugs are not related to the occurrence of cancer, even as a complementary therapy of tumor treatment. To resolve the dispute, numerous recent mechanistic studies using animal models have tried to find the causal link between cancer and different anti-hypertensive drugs. However, the results were often contradictory. Such uncertainties have taken a toll on hypertensive patients. In this review, we will summarize advances of longitudinal studies in the association between anti-hypertensive drugs and related tumor risks that have helped to move the field forward from associative to causative conclusions, in hope of providing a reference for more rigorous and evidence-based clinical research on the topic to guide the clinical decision making.

## Introduction

About 37% of adults in the world suffer from hypertension (Cheungpasitporn et al., 2016; Maharjan, 2018). In addition to healthy lifestyle adjustment, environment (Pranata et al., 2020), long-term oral anti-hypertensive drugs are an important and necessary measure to control blood pressure and reduce the occurrence and death of cardiovascular disease (CVD) As one of the important chronic non-infectious diseases, cancer is out of control of cell growth and basically incurable, which seriously affects people's survival and quality of life (Dong et al., 2018). Therefore, the hypothesis that long-term oral anti-hypertensive drugs may increase the risk of cancer has caused widespread anxiety and fear among hypertensive patients. In 1976, for the first time, a national case-control study in Finland focused on the association between anti-hypertensive drugs and breast cancer was performed. However, no firm conclusions were reached because of cardiopathy, treatment methods, and the effect of breast cancer on outcomes (Aromaa et al., 1976). Subsequently, many investigated the correlation of anti-hypertensive drugs and cancer. The researchers conducted many systematic reviews and meta-analyses regarding various perspectives, especially from the aspects of tumor types (Xie et al., 2020) and anti-hypertensive drug types (Zhao et al., 2016; Datzmann et al., 2019; Asgharzadeh et al., 2020b). Unfortunately, there was no clear answer to eliminate these anxieties; for example, Sipahi et al. (Sipahi et al., 2010) showed angiotensin receptor blockers (ARBs) were moderately associated with cancer while Y.T. Zhao (Zhao et al., 2016) got a controversial result. With this background in mind, this review aimed to analyze the latest studies, existing literature, systematic reviews, and meta-analyses to explore the association between different oral anti-hypertensive drugs and cancer. It is hoped that the conclusions provide a reference for future studies and clinical practices, as well as appropriately alleviate anxiety in patients with hypertension.

## Anti-Hypertensive Drugs Affect Cancer Progression Through Multiple Mechanisms

Hypertension may be an independent risk factor for cancer (Seretis et al., 2019; Xuan et al., 2020), and those two diseases share the same risk factors, such as age, obesity, smoking, alcohol consumption, living environment, as well as social and psychological stress. However, the incidence of cancer is different among patients with hypertension. Regarding the existing evidence, it is believed that there has been an inherent relationship between anti-hypertensive drugs and cancer.

The renin-angiotensin system (RAS) not only plays an important role in CVD, but is also expressed in a variety of cancer environments. It promotes tumor angiogenesis and development (Feng et al., 2010), as well as cancer invasion and metastasis (Uemura et al., 2008). Kosugi et al. (Kosugi et al., 2007) fed candesartan, an ARB, to nude mice of bladder cancer xenograft model and found that candesartan had significantly lower expression of vascular endothelial growth factor (VEGF) in mice; moreover, they showed that the tumor volume was half than that of paclitaxel treatment. Wang et al. (Wang et al., 2008) also found that angiotensin-converting enzyme inhibitors (ACEIs) and ARBs could inhibit the growth and angiogenesis of gastric cancer tumor models. Similarly, Nakamura et al. (Nakamura et al., 2018), Regan et al. (Regan et al., 2019), and Rasha et al. (Rasha et al., 2020) found that the RAS system was involved in the regulation of certain cancer tumor microenvironments. For example, captopril reduces the expression of pro-inflammatory cytokines in breast cancer cell lines and ARB has a certain blocking effect on the immune suppression tumor microenvironment of colon cancer involved by RAS. Matsui et al. (Matsui et al., 2019) proved that Telmisartan (ARB) inhibited cell proliferation and tumor growth of esophageal squamous cell carcinoma by inducing cell cycle arrest. In a recent study, candesartan was shown to significantly sensitize human lung adenocarcinoma cells to tumor necrosis factor-related apoptosis-inducing ligand (TRAIL)-mediated apoptosis by targeting TRAIL-DR5 (Rasheduzzaman and Park, 2018). These findings provide new ideas to overcome the drug resistance of cancer. Therefore, the RAS system involves complex mechanisms in the development of cancer. In addition, the role of ARB/ACEI in cancer requires more relevant preclinical studies.

The role of beta-blockers (BBs) in cancer is mainly the regulation of neurotransmitters. Kun Guo et al. (Guo et al., 2009) revealed that norepinephrine is one of the signal substances present in the tumor environment, which promotes cancer metastasis. It is worth mentioning that the BB propranolol can inhibit this effect. Some studies have found that β-adrenergic receptors (β-ARs) stimulate the proliferation and migration of human pancreatic ductal cancer cells *in vitro* through cAMP-dependent signal transduction (Askari et al., 2005) and propranolol may prevent cAMP-dependent intracellular signal transduction to inhibit this effect (Al-Wadei et al., 2009). A similar inhibitory effect of β-ARs has also been found in angiosarcoma (Amaya et al., 2018). The studies by Xie et al. (Xie et al., 2019), Montoya et al. (Montoya et al., 2019), and Maiko et al. (Sasaki et al., 2019) showed that BBs inhibited the occurrence and development of cancer by regulating gene expressions. Daher et al. (Daher et al., 2019) and Inderberg et al. (Inderberg and Wälchli, 2020) also found that BBs regulated the effect of inflammation on tumors. In general, BBs, such as ACEIs/ARBs, regulate their occurrence and development through multiple channels; nonetheless, more mechanisms will be discovered in the future.

Calcium Channel Blockers（CCBs）and diuretics also have some cancer-related mechanisms. In 1984, Tsuruo et al. (Tsuruo, 1984) found that calcium regulators might increase the level of anti-tumor cells and reduce anti-tumor resistance by inhibiting the delivery of anti-tumor drugs from cancer cells. Recent studies have shown that CCBs reduce the ball formation, viability and proliferation of ovarian cancer stem cells (CSCs), and induce their apoptosis (Lee et al., 2020). Moreover, recent studies have also suggested that the traditional diuretic spironolactone might increase the death and inhibit cell growth of cancer cells treated with gemcitabine and osimertinibe mainly by regulating the expression of the anti-apoptotic protein surviving (Sanomachi et al., 2019).

Obviously, studies on the mechanisms have shown that anti-hypertensive drugs are beneficial in cancer. However, the results of the clinical studies are not consistent with these findings. We will discuss the relationship between anti-hypertensive drugs and tumors from clinical studies.

## The Association Between Anti-Hypertensive Drugs And Cancer Risk

Coleman C et al. (Coleman et al., 2008) performed a meta-analysis on 27 randomized controlled studies (RCT), which included 56 treatment groups, enrolled 126, 137 patients, and identified 5,868 cancers. During follow-up, the results showed that the common anti-hypertensive drugs were not associated with cancer. In 2010, Sipahi et al. (Sipahi et al., 2010) published a meta-analysis in the LANCET including five RCTs involving 61,590 patients, with a follow-up > 1 year. They showed that ARBs were moderately associated with cancer [RR 1.08; 95% confidence interval (95% CI) 1.01–1.15; *p* = 0.016], which was disputed by the researchers. Three studies only included the RCTs (Collaboration, 2011; Sipahi et al., 2011; Zhao et al., 2016) showed that ARBs and ACEIs did not increase the risk of overall or site-specific cancers. The result was consistent with the finding obtained from the meta-analysis of large observational studies (Yang et al., 2015), the latest meta-analysis, and systematic evaluation of high-quality RCTs (Datzmann et al., 2019). Although a study has suggested that combinations of ACEIs and ARBs might increase the RR (Relative Risk) by 10% (Bangalore et al., 2011), the combination of those two drug species is rare in clinical practice. As a result, the association between ARBs/ACEIs and cancer is controversial.

A retrospective cohort with an average follow-up of 10 years suggested that BBs were associated with a decreased level of cancer risk (RR 0.51; 95% CI 0.29–0.90; *p* = 0.02) (Algazi et al., 2004). Monami et al. (Monami et al., 2013) explored the association between BBs and cancer and revealed that BBs could reduce the risk of cancer (Hazard ratio (HR) 0.33; 95% CI 0.13–0.83; *p* = 0.019). Furthermore, Bangalore et al. (Bangalore et al., 2011) conducted a meta-analysis on 70 RCTs involving 324,168 patients and follow-up for at least 1 year. According to the result, there was no data that any single anti-hypertensive drugs increased RR of cancer by 5–10%. However, considering the lack of subgroup analysis of cancer specificity and specific anti-hypertensive drugs, the influence of anti-hypertensive drugs with longer time and larger dosage stays unknown. The mean follow-up duration was from 3 to 5 years. It should be noted that all these limitations exist in this study.

It is known that tumors in different sites have their own specific internal and external pathogenic mechanisms and risk factors. For example, β-AR have been identified in colorectal, lung, breast, and prostate cancer cell lines (Saad et al., 2020), while AT1 receptors are more expressed in breast, laryngeal, pancreatic, and choriocarcinoma cell lines (Uemura et al., 2008). Similarly, same anti-hypertensive drugs may affect the different pathological subtypes and stages of tumors in the same site differently. Based on this hypothesis, in this review, we aimed to explore the relationship between anti-hypertensive drugs and cancer risk focusing on different cancer types. We listed the systematic reviews and meta-analyses of antihypertensive drugs and cancer risk and cancer-related deaths to show our theoretical basis (Table 1).

**TABLE 1:** The systematic reviews and meta-analyses of antihypertensive drugs and cancer risk and cancer-related deaths.

Title	Year	Research type included	Research Quantum	Participants	Anti-hypertensive drugs	*Cancer* types	*Cancer* risk [OR/RR (95%CI)]	Cancer-related deaths [OR/RR (95%CI)]
(Carrao et al., 2007)	2007	Case-control, cohort studies	18	—	Uncategorized	Renal-cell cancer	Hypertension (1.62, 1.24–2.12), diuretics (1.43.1.12–1.83) and no diuretics (1.51; 1.21–1.87)	—
(Coleman et al., 2008)	2008	RCTs	27	126,137	Uncategorized	Uncategorized	ACEIs (0.99, 0.80–1.24), ARBs (1.12, 0.87–1.47), *ß* blockers (1.00, 0.78–1.32), diuretics (0.94, 0.73–1.19), CCBs (0.95, 0.79–1.13)	—
(Sipahi et al., 2010)	2010	RCTs	5	61,590	ARBs	Uncategorized	1.08, 1.01–1.15	—
(Bangalore et al., 2011)	2011	RCTs	70	324,168	Uncategorized	Uncategorized	ARBs (1·01.0·93–1·09), ACEi (1·00, 0·92–1·09), *ß* blockers (0·97, 0·88–1·07), CCBs (1·05, 0·96–1·13), diuretics (1·00, 0·90–1·11), ACEi plus ARBs (1·14, 1·02–1·28)	ARBs (1·00, 0·87–1·15), ACEi (0·95, 0·81–1·10), *ß* blockers (0·93, 0·80–1·08), CCBs (0·96, 0·82–1·11), diuretics (0·98, 0·84–1·13), other controls (1·08, 0·78–1·46), and ACEi plus ARBs (1·10, 0·90–1·32)
(Sipahi et al., 2011)	2011	RCTs	14	61,774	ACEIs	Uncategorized and gastrointestinal cancers	Uncategorized (1.01, 0.95–1.07), GI cancer (1.09, 0.88–1.35)	1.00, 0.88–1.13
(Monami et al., 2013)	2013	RCTs	9	1,340	β blockers	Uncategorized	0.33,0.13–0.83	—
(Chen et al., 2014)	2014	Observational studies	11	—	CCBs	Breast cancer	1.11, 0.93–1.33	—
(Zhang et al., 2015)	2015	Case-control studies, cohort studies	8	298,000	ARBs	Lung cancer	0.81, 0.69–0.94	—
(Dai et al., 2015)	2015	Observational studies	11	≥113,048	ARBs, ACEIs	Colorectal cancer	6% decreased, 0.89–0.98, case-control studies (6% decreased, 0.90–0.99)	0.81, 0.60–1.09
(Thiele et al., 2015)	2015	RCTs	23	2,618	β blockers	Hepatocellular carcinoma	−0.026, −120I.052- −0.001	-0.011, -0.040–0.017
(Yang et al., 2015)	2015	Large observational studies	10	4,020,138	ARBs	Uncategorized and lung cancers	Uncategorized (0.87, 0.75–1.01), lung cancers (0.81, 0.69–0.94)	—
(Zhao et al., 2016)	2016	RCTs	19	148,334	ARBs	Uncategorized	1.08, 1.00–1.18	—
(Qian et al., 2017)	2016	Original research	8	1,994,880	ARBs	Breast cancer	0.93.0.81–1.06	—
(Mao et al., 2016)	2016	Cohort studies	9	20,267	ARBs	Prostate cancer	0.92, 0.87–0.98	—
(Shen et al., 2016)	2016	RCTs, observational studies	31	3,957,725	ARBs, ACEIs	Uncategorized	Observational studies (0.82, 0.73–0.93), RCTs (1.00, 0.92–1.08), lung cancer (0.85, 0.75–0.97)	Observational studies (0.71.0.55–0.93), randomized controlled trials (0.99, 0.89–1.09)
(Wright et al., 2017)	2017	Original research	29	—	CCBs	Breast cancer	1.09, 1.03–1.15	—
(Cao et al., 2018)	2018	Observational studies	21	—	Uncategorized	Prostate cancer	ARBs(1.09, 0.97–1.21), CCBs (1.08, 1–1.16), diuretic (1.09, 0.95–1.25), antiadrenergic agents (1.22, 0.76–1.96)	—
(Gandini et al., 2018)	2018	Independent studies	19	—	Uncategorized	Skin cancer	CCBs (1.14, 1.07–1.21), *ß* blockers (1.21, 1.05–1.40)	—
(Tang et al., 2018)	2018	Observational studies	10	—	Uncategorized	Basal cell carcinoma (BCC) and squamous cell carcinoma (SCC)	Diuretic [ BCC (1.10, 1.01–1.20), SCC (1.40, 1.19–1.66)], *ß* blockers [BCC(1.09, 1.04–1.15)], CCBs [BCC (1.15, 1.09–1.21)]], ACEIs/ARBs [ BCC (0.53, 0.39–0.71), SCC (0.58, 0.42–0.80)	—
(Thakur et al., 2018)	2018	Randomized controlled trials, open prospective studies, pilot studies, and retrospective observations	17	—	CCBs	Breast cancer (BCa) and prostate cancer (PCa)	Bca (1.14.1.02–1.27), pca (1.12, 0.94–1.35)	—
(Rotshild et al., 2018)	2018	Observational studies	10	38,758	CCBs	Lung cancer	1.15, 1.01–1.32	—
(Shin et al., 2019)	2019	Observational studies	9	—	Diuretics	Skin cancers	Squamous cell carcinoma (1.86, 1.23–2.80), basal cell carcinoma (1.19, 1.02–1.38), melanoma (1.14, 1.01–1.29)	—
(Datzmann et al., 2019)	2019	RCTs, observational studies	12	—	ARBs	Uncategorized	RCTs (1.02, 0.87–1.19)	—
(Chen et al., 2020)	2020	Observational studies	16	2,847,597	ARBs, ACEIs	Colorectal cancer	0.86, 0.78–0.93	0.80, 0.66–0.98
(Xie et al., 2020)	2020	Case-control studies, cohort studies	31	3,352,264	Uncategorized	Kidney and bladder cancer	1.45, 1.20–1.75	—

### Respiratory Cancers

According to the results of two large retrospective cohort (438,728 individuals and 1,229,902 patients) studies, ARBs had no association with the risk of lung cancer even the protective factors (Pasternak et al., 2011; Rao et al., 2013). The meta-analysis of large observational trials (Yang et al., 2015) involving 3,827,109 patients from six retrospective cohort studies and 193,029 cases of four case-control studies suggested that ARBs might reduce the risk of lung cancer (RR 0.81; 95% CI 0.69–0.94). Zhang et al. (Zhang et al., 2015) also suggested that ARBs might be related to the reduction of lung cancer risk in the meta-analysis involving 298,000 subjects. In the subgroup analysis, a correlation was observed between race and duration. However, ARBs did not show a risk-reduction conclusion in Caucasians over 5 years. In a retrospective cohort study of 992,061 patients on anti-hypertensive therapy (Hicks et al., 2018) with a mean follow-up of 6.4 years, it was found that ACEIs duration over 5 years significantly increased the risk of lung cancer (HR 1.22; 95% CI 1.06–1.40). The researchers also noted that confounding factors adjustment and lung cancer subtype analysis in this cohort might lead to different correlations.

### Gastrointestinal Cancers

Sipahi et al. (Sipahi et al., 2011) analyzed the association between ACEIs and gastrointestinal cancer in the meta-analysis of 14 RCTs and found that ACEI was not associated with the risk of gastrointestinal cancer（RR 1.01; 95% CI 0.95–1.07; *p* = 0.78）. In the meta-analysis and systematic evaluation of 11 observational studies (Dai et al., 2015), ACEIs/ARBs were found to reduce the risk of colorectal cancer by 6%. A recent meta-analysis of 16 studies involving 2,847,597 participants (Chen et al., 2020) found that RAS inhibitor was associated with a reduced risk of colorectal cancer (RR 0.86; 95% CI 0.78–0.93) and mortality rate (RR 0.80; 95% CI 0.66–0.98). In addition, a sustained increase in RAS inhibitor use per year was associated with a 6% reduction in colorectal cancer risk.

In the same vein, the systematic reviews conducted by Barone et al. (Barone et al., 2019) and Asgharzadeh et al. (Asgharzadeh et al., 2020b) have shown that RAS inhibitors have beneficial effects on hepatocellular carcinoma. Thiele et al. (Thiele et al., 2015) conducted a meta-analysis of RCTs and found that non-selective BBs might prevent the risk of liver cancer in patients with cirrhosis (risk difference −0.026; 95% CI-0.052 to −0.001). The results of a case-control study conducted by Saad et al. (Saad et al., 2020) on patients who were followed-up for more than 2 years showed that long-term use of β-blockers might reduce the risk of pancreatic cancer (OR 0.75; 95% CI 0.57–1.00; *p* = 0.05).

The general conclusion is that RAS system inhibitors and selective BBs have protective effects on gastrointestinal cancers. Relevant studies are still flawed. For example, the included studies may not use the occurrence of cancer as the outcome indicator. Moreover, the treatment process, drug dosage, and different sample sizes have impacts on the research results. Since most large-scale studies currently use electronic health databases, the lack of specific information on tumor pathological tissues is also an important reason for the research limitations.

### Genitourinary System Cancers

As early as 1999, Grossman et al.et al (Grossman et al., 1999). found that long-term use of diuretics might be associated with renal cell carcinoma (RCC) in a meta-analysis involving 1,226,229 participants in 12 studies. The risk was found to be more than doubled, and the result was more prominent in females. Subsequently, in 2007, a meta-analysis of 18 studies further established the association between anti-hypertensive drugs and renal cells (Corrao et al., 2007) by showing that both hypertension and diuretic therapy were risk factors for renal cell carcinoma with significant effects on females. In this study, diuretics were significantly associated with RCC after adjustment for obesity, smoking, and other anti-hypertensive treatments. However, the researchers remained skeptical of the study due to the publication bias and existing evidence-based treatments.

The latest meta-analysis and systematic review (Xie et al., 2020) concluded that each class of anti-hypertensive drugs was associated with renal cancer (RR 1.45; 95% CI 1.20–1.75), while ARBs were associated with an increased risk of bladder cancer (RR 1.07; 95% CI 1.03–1.11). Although the conclusions were adjusted for factors, such as blood pressure and smoking, most of the included studies were observational and presented significant heterogeneity. Researchers are also investigating prostate cancer. A meta-analysis and systematic review conducted by Mao et al. (Mao et al., 2016) showed that ARBs could reduce the risk of prostate cancer (RR 0.92; 95% CI 0.87–0.98). Moreover, the latest meta-analysis including 12 cohort studies (Cao et al., 2018) concluded that common anti-hypertensive agents (i.e., ACEIs, ARBs, BBs, and diuretics) had no significant relationship with prostate cancer risk, whereas CCBs might increase the risk (RR 1.08; 95% CI 1–1.16).

In a meta-analysis performed by Qian et al. (Qian et al., 2017) involving 1,994,880 people, the use of ARBs reduced the risk of breast cancer in Asians (OR 0.62; 95% CI 0.53–0.73; I^2^ = 0%). However, it increased the risk in Caucasians (OR 1.08; 95% CI 1.02–1.13; I^2^ = 28%). In the meta-analysis of 11 observational studies, CHEN et al. (Chen et al., 2014) discussed the association between CCBs and breast cancer and concluded that there was no evidence that CCBs caused an increased risk of breast cancer (OR 1.11; 95% CI 0.93–1.33). However, the short-effect of CCBs preparations might be positively correlated with its occurrence (OR 1.88; 95%CI 1.37–2.60). The following studies also found no association between CCBs and breast cancer (Wright et al., 2017). Similarly, the findings of these studies are weak and lacking a more rigorously designed trial to support the findings. For example, large samples and long duration randomized controlled trials with considerations of drug dosages and medications for comorbidities are needed.

### Other Cancers

Diuretics have photosensitive properties. A population-based case-control study in Denmark (Jensen et al., 2008) firstly found that photosensitive diuretics could cause squamous cell carcinoma (IRR 1.79; 95% CI 1.45–2.21) and melanoma (IRR 1.43; 95% CI 1.09–1.88). Ruiter et al. (Ruiter et al., 2010) found in a prospective Caucasian cohort study that long-term cumulative use of high-dose diuretics was associated with an increased risk of diagnosing basal cell carcinoma (HR 1.6; 95% CI 1.1–2.4), especially in patients who were more prone to sunburn. The latest meta-analysis involving nine observational studies (Shin et al., 2019) found that the use of thiazide diuretics might be associated with an increased risk of skin cancer, especially with squamous cell carcinoma (adjusted odds ratio (aOR) 1.86; 95% CI 1.23–2.80). Bendinelli et al. (Bendinelli et al., 2019) and Garrido et al. (Garrido and Borges-Costa, 2020) also reached a consistent conclusion in this regard. According to a meta-analysis conducted by Gandini et al. (Gandini et al., 2018) involving 77,622 cases, no association between thiazide diuretics, ACEIs, or ARBs and skin cancer risk was found, while CCBs and BBs were associated with an increased risk. In a recent large national case-control study in Denmark, it was found that the use of hydrochlorothiazide was associated with a greatly increased risk of basal cell carcinoma (OR 1.29; 95% CI 1.23–1.35), especially squamous cell carcinoma (OR 3.98; 95% CI 3.68–4.31) (Pedersen et al., 2018). It should be noted that the existing evidence may be controversial, and the included studies were accompanied by methodological limitations. Therefore, there is a need for more carefully designed observational studies to find the answer. When administering anti-hypertensive drugs to patients with hypertension, physicians need to minimize the use and combination of photosensitive diuretics. If those diuretics are inevitable, the patients should be advised to avoid direct sunlight. There are relatively few studies on the association of anti-hypertensive drugs with head and neck cancers. Recently, Kim et al. (Kim et al., 2019) found that there was no relationship of BBs with head and neck cancers through a case-control study involving 12,127 patients (OR 1.18; 95% CI 1.105–1.26).

As we mentioned above, the results of clinical studies are inconsistent with preclinical studies. Gap is existing between preclinical studies and clinical studies, rigorously designed trails and a simplified system may help to provide insights of some aspects of the mechanisms, since cell line and animal models are limited in their ability to mimic the extremely complex conditions of cancer and hypertension.

### Anti-Hypertensive Drugs Have the Potential to Become Adjuvant Therapy Drugs for Cancer

The molecular targets of anti-hypertensive drugs were found to play important roles on tumor development, migration, recurrence, and drug resistance. As a result, anti-hypertensive drugs are also used for the combinational treatment of cancer and the improvement of patient prognosis. The RAS system plays a crucial role in the development and metastasis of cancer, and researchers have used RAS inhibitors to assess the prognosis of cancer patients. To evaluate the potential evidence of RASB for cancer recurrence and survival, Song et al. (Song et al., 2017) conducted a systematic review of 11 studies in 2017. They found that the use of ACEIs or ARBs in cancer patients can reduce the risk of cancer recurrence and death by 40 and 25%, respectively. Systematic reviews by MC Menamin et al. (Mc Menamin Ú et al., 2012) and Li et al. (Li et al., 2017) also suggested that RAS inhibitors could improve cancer prognoses, such as improved survival rate and reduced risk of disease progression. On the other hand, because of the cardiotoxicity caused by chemotherapy drugs through the RAS system (Sobczuk et al., 2020), RAS inhibitors appears to moderately reduce the chemotherapy-related cardiotoxicity associated with cancer (Fang et al., 2020). When RAS inhibitors are used in certain site-specific cancer, for example in breast cancer, there was no evidence to support better disease-free survival and overall survival (Raimondi et al., 2016). However, it can improve survival and reduce mortality in kidney cancer (Asgharzadeh et al., 2020a), and show beneficial outcomes in gastrointestinal cancer (Zhou et al., 2020). In response to partial incoherence, a systematic review and meta-analysis by Shen et al. (Shen et al., 2016) observed significant benefits of RAS inhibitors in case-control and cohort studies, whereas they were attenuated in randomized controlled trials. It is worth mentioning that clinical design, cancer type, and follow-up duration may influence the clinical outcomes.

The presence of β-ARs and norepinephrine play an important role in tumor growth and metastasis; moreover, psychosocial stressors also directly stimulate tumor growth mainly through sympathetic nerves (Mravec et al., 2018). In 2013, a meta-analysis (Monami et al., 2013) proved that BBs seemed to be a possible way to counter the risk of cancer development. Zhong et al. (Zhong et al., 2016) suggested that the use of BBs after a cancer diagnosis may lead to better overall survival of cancer. Charak et al. (Charak et al., 2018) also suggested that BBs were associated with better specific cancer survival. Furthermore, the results of a systematic review of eight high-quality studies (Barbosa et al., 2018) indicated that BBs had potential protective effects on the heart. Additionally, for early-stage cancer treated with surgery, BBs could prolong the survival period (Choi et al., 2014).

For site-specific cancers, the focus was mainly on the role of BBs in breast cancer. Through systematic review and meta-analysis of published literature, it was confirmed that BBs could significantly reduce the risk of mortality (Childers et al., 2015), recurrence (Zhao et al., 2018), and cardiotoxicity treated with anthracyclines in breast cancer (Shah et al., 2019). There are also relevant studies showing that BBs have no significant relationship with breast cancer survival, recurrence, and other aspects (Kim et al., 2017). Moreover, it is believed that the beneficial effect of BBs on breast cancer may be based on the immortal time deviation (Weberpals et al., 2016). However, due to the weakness of overall research quality, the beneficial conclusion seems unreliable (Spini et al., 2019). The BBs have been currently used in other cancer types, such as glioblastoma (Tewarie et al., 2020), lung cancer (Coelho et al., 2020), and ovarian cancer (Majidi et al., 2020). However, its role in cancer therapy is difficult to determine because of the quality of researches. The CCBs do not appear to improve prognosis in cancer therapy in the current systematic reviews (Sun et al., 2016; Wright et al., 2017); however, the conclusions remain uncertain.

## Future Directions of Anti-Hypertensive Drugs and Cancer

In view of the common limitations of current studies, we propose in this section the future directions of antihypertensive drugs and cancer, including cancer risk and adjuvant therapy.

Regarding the perspective of anti-hypertensive drugs, a great number of studies were focused on the use of common five types of anti-hypertensive drugs, such as ACEIs, ARBs, BBs, CCBs, and diuretics. Although direct vasodilators, centrally acting drugs and renin inhibitors are not first-line recommendations, many hypertensive patients are still taking them for a long time. There is also evidence that folic acid can help hypertensive patients with high homocysteine ​​to control blood pressure (Singh et al., 2019). Moreover, compound monolithic preparations combined with anti-hypertensive drugs and folic acid are utilized in clinical practices. Unfortunately, there are few or no clinical studies to focus on the association between the above-mentioned drugs and cancer. A few preclinical studies have found that α-blocker can inhibit the growth of certain tumor cells (Vázquez et al., 2006; Lin et al., 2007; Suzuki et al., 2020). To our knowledge, only five studies investigated the association of the above-mentioned drugs (anti-hypertensive drugs and folic acid for hypertensive patients) with cancer risk, even fewer for adjuvant therapy for cancer. Out of these studies, four studies evaluated the association of reserpine with cancer risk from 1975 to 1999 (Laska et al., 1975; Lilienfeld et al., 1976; Curb et al., 1982; Ogihara et al., 1999). The other investigated the correlation between folic acid and cancer in 2017 (Qin et al., 2017). In addition, the data revealed that 75% of hypertensive patients require a combination of drugs to control blood pressure (Gradman et al., 2010). Whether multiple anti-hypertensive drugs will have different effects on the body of cancer at the same time is lacking in current studies. The majority of patients with hypertension have a comorbidity of multiple other diseases. A recent study showed that 55.7–62.1% of adults at 18–64 years were living with one or more chronic conditions (Chapel et al., 2017). Therefore, people with hypertension need to take anti-hypertensive drugs for a long time, they may also take different types of hypoglycemic, lipid-lowering, and antiplatelet drugs. Whether these drugs and anti-hypertensive drugs work together will lead to different outcomes is currently unclear. A recent study on the association between cardiovascular comorbidities and epithelial ovarian cancer found that high blood pressure increased the risk of epithelial ovarian cancer, whereas diabetes and hyperlipidemia reduced the risk. Therefore, the author proposed that anti-hypertensive drugs, antidiabetic drugs, or lipid-lowering drugs may have an impact on cancer risk (Staples et al., 2020). In addition, current studies paid little attention to specific components in anti-hypertensive drugs. The auxiliary components and impurities in the production of the anti-hypertensive drugs also need to be investigated in future studies. Berrido et al. (Berrido and Byrd, 2020) considered that the nitrite in anti-hypertensive drugs might be an important cause of cancer. Finally, the influence of the dosage of anti-hypertensive drugs should be considered in the studies, which is a widespread limitation in the current studies.

Regarding the study design, the majority of the current studies are observational, and confounding factors are poorly controlled. In particular, most large-sample studies come from electronic health databases, which have incomplete information on risk factors and family history. This limits the reliability of the research results. In 2019, a national study of 12,127 patients in South Korea investigated the association between BBs and cancer. However, the conclusion was influenced by unreliable data on smoking history (Kim et al., 2019). Htoo et al. (Htoo et al., 2019) observed 111,533 individuals using ACEI/ARB and found no association between ACEI/ARB and short-term rectal cancer. In the aforementioned study, the elderly’s body mass index (BMI), smoking, alcohol, diet, and functional restrictions were unmeasured and confused. Moreover, most observational studies are currently retrospective, and recall bias is inevitable. Large-scale prospective cohort studies and randomized controlled trials are currently important research methods to explore the exact relationship between anti-hypertensive drugs and tumors. Furthermore, the choice of patients will affect the results of a study. Age, gender, related family history, and comorbidities are particularly relevant for a certain type of cancer. According to the purpose of the study, appropriate inclusion and exclusion criteria will lead to more reliable results. Since hypertensive patients may have different types of anti-hypertensive drugs during the long-term course of the disease, the results obtained only based on the prescription data or patient surveys are biased. Therefore, the selection of new hypertensive patients and the design of prospective studies can effectively control the bias. In a study conducted by Htoo et al. (Htoo et al., 2019), selected new users of anti-hypertensive drugs were selected to build a cohort. Although the study still has certain shortcomings and the current average follow-up time is only 2.2 years, the new user design is an advantage that many previous studies did not have. A study also showed BBs may lead to a better overall survival of cancer after a cancer diagnosis (Zhong et al., 2016). A retrospective cohort study (NCT04334824) of a large sample and new user design on hydrochlorothiazide and skin cancer in Canada is also worthy of subsequent attention.

Additionally, patient medication compliance needs to be taken into consideration. Studies have found that about half of the patients stopped anti-hypertensive drugs after one year (Newby et al., 2006), and the results of the study would be seriously affected by medication compliance.

Finally, since cancer is a process of long-term progress, current studies did not include sufficient follow-up time. There are no large-scale studies with more than 10 years of follow-up and rigorous design. After paying more attention to the relationship between anti-hypertensive drugs and cancer, we believe that more large-scale cohort studies should emphasize more on this issue.

In general, the current relationship between antihypertensive drugs and cancer needs further research, including preclinical and clinical research, cancer risk, and adjuvant therapy.

## Conclusion

Hypertension is a major public health problem with high prevalence. Hypertensive patients require long-term oral anti-hypertensive drugs. Therefore, it is necessary to clarify the relationship between anti-hypertensive drugs and cancer. Our review summarized the latest studies and has considered the cancer sites, the types of anti-hypertensive drugs, and the credibility of evidence to expound the relationship between anti-hypertensive drugs and cancer from different standpoints. It is believed that the relationship between anti-hypertensive drugs and cancer risk and prognosis is not clear yet. However, certain conclusions from current studies can guide clinical practice, such as instructing patients who use photosensitive diuretics to reduce UV exposure. Future studies need to consider multiple perspectives comprehensively, such as the type of cancers, the selection and dosage of drugs, the selection of patients, the study design, and the research methods to draw more reliable conclusions.

## Author Contributions

YZ and XL designed the review. RY contributed to manuscript writing, revised, and supervised the project. YY, CH and LL contributed to literature searches and to preparing the manuscript draft. All authors approved the manuscript.

## Funding

This study is supported by the grant of National Clinical Research Center for Geriatrics, West China Hospital, Sichuan University (No. Z20191009), the grant of Department of Science and Technology in Sichuan Provincial (No. 20SYSX0293).

## Conflict of Interest

The authors declare that the research was conducted in the absence of any commercial or financial relationships that could be construed as a potential conflict of interest.
